# Supplementary material for: Trending Towards Safer Breast Cancer Surgeries? Examining Acute Complication Rates from A 13-Year NSQIP Analysis
Source: Cancers (Basel). 2019 Feb 21;11(2):253. doi: 10.3390/cancers11020253 (PMC6407023; doi:10.3390/cancers11020253)
Supplement: Supplementary file 1 [file cancers-11-00253-s001.pdf]

Supplementary Materials: Trending Towards Safer Breast Cancer Surgeries? Examining Acute Complication Rates from A 13-Year NSQIP Analysis

Michael M. Jonczyk, Jolie Jean, Roger Graham and Abhishek Chatterjee

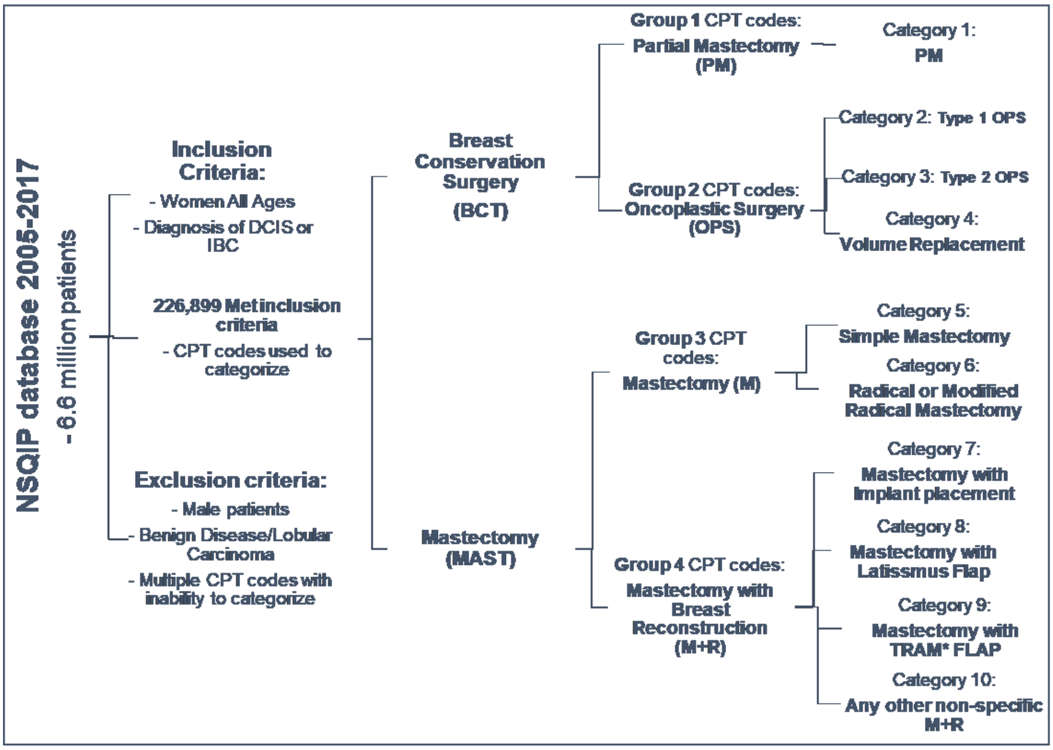

Figure S1. Methods Schematic. * TRAM Flap: transverse abdominis myocutaneous flap.

Table S1. CPT Codes use to Categorize Breast Cancer Intervention.

Group	CPT Code	Category (CG)	Group	CPT Code	Category (CG)
**Group 1: Breast Conservative Surgery**	19160	1	**Group 2: Oncoplastic Surgery (Any Group 1 CPT Code plus one of these)**	14000	2
	19162			14001	2
	19301			14300-01	2
	19302			14302	2
**Group 3: Mastectomy**	19180	5		19366	3
	19182	5		19316	3
	19303	5		19318	3
	19304	5		Any Breast Reconstruction CPT Code	4 **
	19240	6	**Breast Reconstruction**	19340, 19342	7 *
	19200	6		19357, 15777	7 *
	19220	6		19361	8 *
	19305	6		19367	9 *
	19306	6		19368	9 *
	19307	6		19369	9 *
**Group 4: Mastectomy + Breast Reconstruction**	Any Group 2 CPT code plus any Breast Reconstruction CPT Code	* Four groups		19364	10 *
				19366	10 *
				15734, 15740, 15757	10

* CG reference to any patient undergoing a Mastectomy with breast reconstruction (Group 4); ** Patients with OPS (CG4) were categorized based on any patient in Group 1 and any corresponding CPT code in the breast reconstruction section

Table S2. Data from NSQIQ database.

Year	Total Participants	Breast Cancer	Exclusion	Inclusion
**2005**	33,930	2366	590	1776
**2006**	118,560	7749	1659	6090
**2007**	211,407	13,411	2734	10,677
**2008**	271,368	15,413	2908	12,505
**2009**	336,190	18,858	3439	15,419
**2010**	363,431	18,792	3375	15,417
**2011**	442,149	19,234	3411	15,823
**2012**	545,071	41,734	22,939	18,795
**2013**	651,490	26,161	3983	22,178
**2014**	750,397	27,808	4275	23,533
**2015**	885,502	30,628	4641	25,987
**2016**	1,000,393	34,162	4975	29,187
**2017**	1,028,713	34,560	5048	29,512
**Total**	6,638,601	290,985	64,059	226,899

All patients in NSQIP database by year and of those patients included in our study.

Table S3A. Unadjusted Logistical Regression.

Patient Demographics	Wound Complications			Infection Complications			Respiratory Complications		
	**OR**	**(95% CI)**	***p*-Value**	**OR**	**(95% CI)**	***p*-Value**	**OR**	**(95% CI)**	***p*-Value**
Surgery Type									
OPS	1.26	(1.05–1.51)	0.014	1.44	(1.08–1.93)	0.015	1.17	(0.57–2.40)	0.677
M	2.45	(2.29–2.63)	<0.0001	2.46	(2.19–2.76)	<0.0001	2.59	(2.00–3.34)	<0.0001
M + R	3.80	(3.31–4.37)	<0.0001	4.12	(3.29–5.16)	<0.0001	2.69	(1.47–4.91)	0.001
M + I	2.17	(2.01–2.35)	<0.0001	3.13	(2.77–3.53)	<0.0001	1.03	(0.72–1.49)	0.855
Age (Young: <60)	1.12	(1.06–1.18)	<0.0001	0.98	(0.90–1.07)	0.702	0.54	(0.43–0.66)	<0.0001
Race									
Black	1.00	(0.91–1.09)	0.924	0.87	(0.76–1.01)	0.062	1.19	(0.87–1.63)	0.265
Asian/Pacific	0.50	(0.42–0.60)	<0.0001	0.46	(0.35–0.61)	<0.0001	0.46	(0.23–0.93)	0.030
Native	1.89	(1.43–2.51)	<0.0001	1.98	(1.29–3.02)	0.002	0.55	(0.08–3.92)	0.551
Pathology (Invasive)	1.20	(1.11–1.28)	<0.0001	1.18	(1.05–1.33)	0.004	1.34	(1.00–1.80)	0.049
Admission Status (Outpatient)	0.52	(0.50–0.55)	<0.0001	0.51	(0.47–0.55)	<0.0001	0.27	(0.22–0.34)	<0.0001
BMI									
Underweight	1.07	(0.85–1.33)	0.571	1.36	(1.00–1.84)	0.050	2.79	(1.63–4.78)	<0.0001
Overweight	1.40	(1.29–1.52)	<0.0001	1.34	(1.18–1.51)	<0.0001	1.12	(0.82–1.52)	0.486
Obese	2.47	(2.30–2.65)	<0.0001	1.95	(1.74–2.17)	<0.0001	1.94	(1.49–2.54)	<0.0001
Diabetic									
Non-Diabetic	0.46	(0.41–0.51)	<0.0001	0.42	(0.36–0.49)	<0.0001	0.39	(0.26–0.57)	<0.0001
Diabetic-Oral	0.68	(0.60–0.77)	<0.0001	0.60	(0.49–0.73)	<0.0001	0.66	(0.42–1.06)	0.084
Pregnancy Status	0.45	(0.06–3.25)	0.432	1.36	(0.19–9.75)	0.762	0.00	(0.00–3E+218)	0.969
Smoking Status Smoker	1.76	(1.65–1.88)	<0.0001	1.36	(1.21–1.52)	<0.0001	1.67	(1.28–2.18)	<0.0001
Smoking Pack Per Day (PPD)									
0–20 PPD	1.29	(0.99–1.68)	0.063	1.23	(0.78–1.93)	0.366	0.35	(0.05–2.49)	0.292
21–50 PPD	1.44	(1.25–1.65)	<0.0001	1.45	(1.15–1.83)	0.002	2.14	(1.34–3.42)	0.001
>100 PPD	1.51	(1.32–1.74)	<0.0001	1.43	(1.13–1.81)	0.003	1.72	(1.03–2.88)	0.039
Steroid Use	1.34	(1.14–1.57)	<0.0001	1.62	(1.28–2.06)	<0.0001	2.05	(1.22–3.44)	0.007
Prior Chemotherapy	1.30	(1.09–1.54)	0.003	1.67	(1.29–2.17)	<0.0001	1.61	(0.88–2.95)	0.124
Prior Radiation Therapy	1.55	(0.89–2.70)	0.124	2.45	(1.15–5.18)	0.020	3.68	(0.91–14.85)	0.068
Recent Weight Loss	2.04	(1.52–2.74)	<0.001	4.03	(2.86–5.66)	<0.0001	5.47	(2.71–11.05)	<0.0001
PMH of Angina	3.23	(1.83–5.70)	<0.0001	1.38	(0.34–5.58)	0.650	7.41	(1.83–30.04)	0.005
PMH of CHF	1.90	(1.30–2.78)	0.001	2.84	(1.72–4.67)	<0.0001	12.04	(6.57–22.06)	<0.0001
PMH of Renal Failure	2.75	(1.00–7.57)	0.051	3.53	(0.86–14.46)	0.080	21.57	(5.25–88.61)	<0.0001
PMH of Bleeding Disorders	1.82	(1.55–2.14)	<0.0001	2.08	(1.64–2.66)	<0.0001	5.55	(3.79–8.13)	<0.0001

PMH of COPD	1.75	(1.55–1.99)	<0.0001	1.87	(1.54–2.27)	<0.0001	4.96	(3.61–6.80)	<0.0001
PMH of Hypertension	1.26	(1.20–1.34)	<0.001	1.30	(1.19–1.41)	<0.0001	2.21	(1.78–2.74)	<0.0001
Recent Pneumonia	5.38	(1.22–23.70)	0.026	0.00	(0.00–1E+110)	0.947	0.00	(0.00–2E+184)	0.970
Dyspnea									
Moderate	1.08	(0.71–1.65)	0.723	1.02	(0.54–1.94)	0.942	0.51	(0.22–1.17)	0.112
None	0.63	(0.41–0.95)	0.028	0.55	(0.29–1.03)	0.060	0.14	(0.06–0.31)	
ASA Class									
2-Mild Disturb	1.74	(1.48–2.03)	0.000	2.05	(1.54–2.74)	<0.0001	1.07	(0.56–2.05)	0.830
3-Severe Disturb	2.61	(2.23–3.06)	<0.0001	3.47	(2.61–4.63)	<0.0001	3.34	(1.77–6.30)	<0.0001
4-Life Threatening	3.28	(2.59–4.15)	<0.0001	6.74	(4.71–9.64)	<0.0001	16.98	(8.50–33.93)	<0.0001
5-Morbid	39.28	(3.54–435.33)	0.003	0.00	(0.00–2E+94)	0.958	0.00	(0.00–Inf)	0.996
Open Wound Infection	2.70	(2.19–3.33)	<0.0001	4.00	(3.04–5.27)	<0.0001	8.60	(5.40–13.69)	<0.0001
Any Operation in Last 30 Days	0.88	(0.70–1.11)	0.283	0.75	(0.49–1.15)	0.186	1.27	(0.60–2.71)	0.530
Operating Time									
1–2 h	1.67	(1.54–1.82)	<0.0001	1.52	(1.32–1.75)	<0.0001	1.23	(0.93–1.64)	0.151
2–3 h	2.22	(2.03–2.43)	<0.0001	2.16	(1.86–2.50)	<0.0001	1.07	(0.76–1.52)	0.684
3–5 h	2.56	(2.34–2.81)	<0.0001	3.00	(2.59–3.47)	<0.0001	1.04	(0.71–1.50)	0.852
5–10 h	3.47	(3.11–3.88)	<0.0001	4.32	(3.65–5.12)	<0.0001	2.13	(1.41–3.21)	<0.0001
10+ h	5.00	(4.05–6.17)	<0.0001	4.72	(3.35–6.64)	<0.0001	8.13	(4.79–13.81)	<0.0001

Table S3B. Unadjusted Logistical Regression.

**Patient Demographics**	**Bleeding Complications**			**Thromboembolic Complications**			**Renal Complications**		
	**OR**	**(95% CI)**	***p*-Value**	**OR**	**(95% CI)**	***p*-Value**	**OR**	**(95% CI)**	***p*-Value**
Surgery Type									
OPS	5.58	(3.59–8.69)	<0.0001	1.19	(0.60–2.36)	0.613	1.25	(0.29–5.33)	0.764
M	17.49	(13.61–22.46)	<0.0001	2.74	(2.15–3.48)	<0.0001	3.41	(2.06–5.66)	<0.0001
M + R	65.57	(49.63–86.65)	<0.0001	11.29	(8.11–15.72)	<0.0001	2.87	(0.86–9.64)	0.087
M + I	9.14	(6.99–11.95)	<0.0001	3.93	(3.07–5.02)	<0.0001	1.13	(0.55–2.35)	0.735
AGE: (Young: <60)	1.51	(1.38–1.66)	<0.0001	0.93	(0.79–1.10)	0.397	0.70	(0.47–1.05)	0.086
Race									
Black	2.24	(1.99–2.52)	<0.0001	1.33	(1.05–1.67)	0.018	2.29	(1.41–3.72)	0.001
Asian/Pacific	1.53	(1.26–1.85)	<0.0001	0.42	(0.24–0.75)	0.003	0.24	(0.03–1.70)	0.152
Native	1.40	(0.77–2.54)	0.272	0.34	(0.05–2.40)	0.278	0.00	(0.00–8E+268)	0.971
Pathology (Invasive)	1.24	(1.10–1.41)	0.001	1.18	(0.95–1.47)	0.130	1.37	(0.78–2.41)	0.277
Admission Status (Outpatient)	0.08	(0.07–0.09)	<0.0001	0.34	(0.28–0.40)	<0.0001	0.27	(0.18–0.41)	<0.0001
BMI									
Underweight	0.82	(0.57–1.17)	0.281	0.60	(0.25–1.48)	0.268	2.51	(0.73–8.62)	0.144
Overweight	1.09	(0.97–1.23)	0.139	1.53	(1.20–1.95)	0.001	1.47	(0.78–2.78)	0.236

Obese	1.24	(1.11–1.38)	<0.0001	2.33	(1.87–2.89)	<0.0001	2.89	(1.66–5.04)	<0.0001
Diabetic									
Non-Diabetic	0.51	(0.43–0.62)	<0.0001	1.18	(0.74–1.89)	0.483	0.14	(0.08–0.25)	<0.0001
Diabetic-Oral	0.60	(0.48–0.76)	<0.0001	1.00	(0.58–1.74)	0.992	0.48	(0.25–0.92)	0.027
Pregnancy Status	1.46	(0.20–10.53)	0.705	0.00	(0.00–1E+131)	0.952	0.952	(0.00–Inf)	0.982
Smoking Status Smoker	0.93	(0.80–1.07)	0.295	0.85	(0.65–1.10)	0.211	1.21	(0.69–2.13)	0.510
Smoking Pack Per Day (PPD)									
0–20 PPD	1.29	(0.72–2.30)	0.394	0.81	(0.30–2.20)	0.684	0.91	(0.12–6.73)	0.930
21–50 PPD	1.03	(0.73–1.47)	0.850	1.25	(0.80–1.96)	0.319	0.74	(0.22–2.42)	0.613
>100 PPD	0.77	(0.51–1.15)	0.203	0.90	(0.53–1.51)	0.684	1.26	(0.49–3.27)	0.631
Steroid Use	2.09	(1.67–2.62)	<0.0001	1.98	(1.31–2.98)	0.001	2.52	(1.02–6.20)	0.044
Prior Chemotherapy	1.49	(1.12–1.99)	0.006	1.14	(0.64–2.02)	0.664	2.71	(1.09–6.75)	0.032
Prior Radiation Therapy	0.74	(0.18–2.98)	0.672	2.40	(0.60–9.69)	0.217	0.00	(4.34e-311–inf)	0.976
Recent Weight Loss	4.48	(3.17–6.33)	<0.0001	1.21	(0.39–3.76)	0.746	18.41	(8.51–39.81)	0.000
PMH of Angina	2.25	(0.72–7.07)	0.164	0.00	(0.00–3E+154)	0.955	0.00	(0.00–Inf)	0.983
PMH of CHF	4.53	(2.95–6.95)	<0.0001	2.54	(0.95–6.82)	0.064	15.99	(5.86–43.64)	<0.0001
PMH of Renal Failure	4.04	(0.99–16.55)	0.052	0.00	(0.00–3E+154)	0.959	80.08	(19.29–332.39)	<0.0001
PMH of Bleeding Disorders	3.57	(2.91–4.38)	<0.0001	2.07	(1.31–3.28)	0.002	5.57	(2.70–11.49)	<0.0001
PMH of COPD	1.20	(0.93–1.54)	0.168	1.42	(0.93–2.15)	0.101	3.56	(1.79–7.07)	<0.0001
PMH of Hypertension	1.14	(1.04–1.25)	0.005	1.13	(0.96–1.33)	0.14	4.31	(2.70–6.90)	<0.0001
Recent Pneumonia	0.00	(0.00–1E+110)	0.948	0.00	(0.00–1E+184)	0.969	0.00	(7.30e-314–inf)	0.983
Dyspnea									
Moderate	0.93	(0.41–2.13)	0.866	0.59	(0.21–1.63)	0.305	5.17E+5	(0.00–Inf)	0.976
None	0.84	(0.37–1.87)	0.664	0.38	(0.14–1.03)	0.057	1155	(0.00–Inf)	0.979
ASA Class									
2-Mild Disturb	1.61	(1.21–2.14)	0.001	1.29	(0.85–1.95)	0.229	1.74	(0.23–13.04)	0.589
3-Severe Disturb	2.96	(2.23–3.93)	<0.0001	1.69	(1.11–2.57)	0.015	11.91	(1.65–85.71)	0.014
4-Life threatening	8.42	(6.00–11.81)	<0.0001	2.05	(1.02–4.10)	0.043	36.92	(4.68–291.52)	0.001
5-Morbid	0.00	(0.00–2E+94)	0.958	0.00	(0.00–Inf)	0.996	0.00	(0.00–Inf)	0.998
Open Wound Infection	6.94	(5.50–8.74)	<0.0001	1.55	(0.69–3.47)	0.286	8.18	(3.32–20.15)	<0.0001
Any Operation in Last 30 Days	0.53	(0.31–0.92)	0.025	2.44	(1.53–3.89)	<0.0001	2.91	(1.05–8.06)	0.040
Operating Time									
1–2 h	2.57	(2.09–3.16)	<0.0001	1.98	(1.44–2.73)	<0.0001	1.39	(0.78–2.49)	0.265
2–3 h	3.65	(2.94–4.53)	<0.0001	3.34	(2.41–4.64)	<0.0001	1.72	(0.91–3.27)	0.095
3–5 h	4.92	(3.98–6.10)	<0.0001	4.69	(3.40–6.47)	<0.0001	1.40	(0.69–2.85)	0.348
5–10 h	20.95	(17.06–25.71)	<0.0001	9.03	(6.43–12.69)	<0.0001	2.24	(0.97–5.20)	0.060
10+ h	88.17	(70.15–110.82)	<0.0001	28.18	(18.57–42.76)	<0.0001	8.27	(2.78–24.61)	<0.0001

Table S3C. Unadjusted Logistical Regression.

**Patient Demographics**	**Cardio Complications**			**Neuro Complications**		
	**OR**	**(95% CI)**	***p*-Value**	**OR**	**(95% CI)**	***p*-Value**
Surgery Type						
OPS	1.25	(0.45–3.48)	0.671	1.81	(0.64–5.15)	0.266
M	2.87	(1.99–4.14)	<0.0001	2.99	(1.92–4.63)	<0.0001
M + R	1.44	(0.45–4.64)	0.544	2.78	(0.98–7.90)	0.056
M + I	0.41	(0.19–0.88)	0.022	0.75	(0.36–1.53)	0.426
Age (Young: <60)	0.26	(0.18–0.38)	<0.0001	0.18	(0.11–0.29)	<0.0001
Race						
Black	0.73	(0.40–1.32)	0.291	1.04	(0.58–1.86)	0.905
Asian/Pacific	0.82	(0.36–1.87)	0.640	0.72	(0.26–1.97)	0.525
Native	2.63	(0.65–10.65)	0.176	3.46	(0.85–14.09)	0.083
Pathology (Invasive)	1.60	(0.99–2.58)	0.056	1.77	(1.00–3.16)	0.052
Admission Status (Outpatient)	0.32	(0.23–0.45)	<0.0001	0.48	(0.33–0.69)	<0.0001
BMI						
Underweight	3.53	(1.76–7.08)	<0.0001	1.43	(0.44–4.72)	0.553
Overweight	1.02	(0.65–1.60)	0.920	1.24	(0.75–2.05)	0.395
Obese	1.44	(0.96–2.14)	0.076	1.45	(0.91–2.30)	0.120
Diabetic						
Non-Diabetic	0.24	(0.15–0.39)	<0.0001	0.22	(0.12–0.37)	<0.0001
Diabetic-Oral	0.43	(0.23–0.81)	0.009	0.49	(0.25–0.98)	0.043
Pregnancy Status	0.00	(0.00–Inf)	0.981	0.00	(0.00–Inf)	0.981
Smoking Status (Smoker)	0.97	(0.60–1.59)	0.919	1.10	(0.64–1.89)	0.732
Smoking Pack Per Day (PPD)						
0–20 PPD	2.23	(0.53–9.46)	0.277	2.05	(0.49–8.66)	0.329
21–50 PPD	1.49	(0.57–3.93)	0.417	1.37	(0.53–3.59)	0.517
>100 PPD	2.46	(1.10–5.50)	0.028	2.26	(1.02–5.02)	0.044
Steroid use	1.93	(0.85–4.36)	0.116	2.15	(0.88–5.27)	0.094
Prior Chemotherapy	0.36	(0.05–2.54)	0.303	0.00	(0.00–Inf)	0.975
Prior Radiation Therapy	0.00	(3.04e-311–6E+300)	0.975	5.83	(0.81–42.03)	0.081
Recent Weight Loss	4.80	(1.53–15.07)	0.007	2.10	(0.29–15.08)	0.459
PMH of Angina	18.94	(4.63–77.51)	0.000	11.70	(1.62–84.67)	0.015
PMH of CHF	2.48	(0.35–17.72)	0.367	6.69	(1.65–27.13)	0.008
PMH of Renal Failure	0.00	(0.00–3E+257)	0.976	0.00	(0.00–4E+257)	0.977
PMH of Bleeding Disorders	6.37	(3.67–11.04)	<0.0001	2.87	(1.17–7.04)	0.021
PMH of COPD	4.74	(2.89–7.75)	<0.0001	3.39	(1.77–6.48)	<0.0001
PMH of Hypertension	4.19	(2.89–6.07)	<0.0001	4.13	(2.69–6.33)	<0.0001
Recent Pneumonia	0.00	(5.13e-314–1E+306)	0.982	0.00	(6.43e-314–2E+306)	0.982
Dyspnea						
Moderate	0.62	(0.15–2.64)	0.521	1.24E+05	(0.00–2E+230)	0.965
None	0.18	(0.04–0.72)	0.015	5.46E+04	(0.00–8E+229)	0.967
ASA Class						
2-Mild Disturb	1.19	(0.37–3.87)	0.769	3.68	(0.50–26.77)	0.199
3-Severe Disturb	5.22	(1.65–16.48)	0.005	11.57	(1.61–83.30)	0.015
4-Life threatening	27.44	(8.15–92.40)	<0.0001	28.70	(3.53–233.33)	0.002
5-Morbid	0.00	(0.00–Inf)	0.998	0.00	(0.00–Inf)	0.998
Open Wound Infection	4.11	(1.52–11.11)	0.005	0.00	(0.00–2E+232)	0.966
Any Operation in Last 30 Days	0.45	(0.06–3.25)	0.430	0.57	(0.08–4.11)	0.578
Operating Time						
1–2 h	0.87	(0.58–1.28)	0.472	1.15	(0.73–1.79)	0.552
2–3 h	0.81	(0.50–1.31)	0.381	0.72	(0.39–1.32)	0.285
3–5 h	0.38	(0.19–0.75)	0.005	0.55	(0.27–1.12)	0.100
5–10 h	1.16	(0.60–2.25)	0.650	0.46	(0.14–1.51)	0.200
10+ h	2.34	(0.73–7.54)	0.154	3.40	(1.04–11.13)	0.043

© 2019 by the authors. Licensee MDPI, Basel, Switzerland. This article is an open access article distributed under the terms and conditions of the Creative Commons Attribution (CC BY) license (http://creativecommons.org/licenses/by/4.0/).
